# Some of the Dangers of an Impure Meat-Supply and Milk-Supply1Read before the Louisiana State Sanitary Association at Baton Rouge, June, 1899.

**Published:** 1900-05

**Authors:** W. H. Dalrymple


### SOME OF THE DANGERS OF AN IMPURE MEAT-SUPPLY AND MILK-SUPPLY.[1]

By W. H. Dalrymple, M.R.C.V.S.

The success of all great reforms is largely dependent upon the education of public opinion. Ignorance is the mighty incubus which retards the advancement of all progressive movements. Science has had to struggle on through the ages against the enormous odds of incredulity, indifference, and superstition; but truth will prevail, and scientific investigation, which is but the searching after truth, although hampered on all sides by those influences which are born of ignorance, superstition, and doubt, is slowly but surely revealing the true nature of things, and must eventually and effectually succeed, but the rate of progress, in the accomplishment of the desired end, will greatly depend upon the acceptance of the revealed truths by the people, which can only be attained by the education of public opinion.

There is no education more valuable in its possession, nor more philanthropic in its aim, than that which has for its object the amelioration of the conditions of our fellowmen. Life without health is an emasculated existence, shorn of everything that goes to make up the pleasure of living; and the individual, the association, or the community of whom it can be said that he or they inaugurated or stood up for reforms that resulted in the preservation of health, or the prolongation of even one human life, has accomplished a work the value of which can hardly be estimated.

[1] Read before the Louisiana State Sanitary Association at Baton Rouge, June, 1899.

What are the purposes and objects of this association? They may be summed up in the following sentence: The conservation of public health and the prolongation of human life and its hope of success lie mainly in being able to place before the people scientific facts along the various branches of sanitation in a way that they can be received and intelligently acted upon, so that by concert of action much-needed reforms may be brought about, disease lessened, death made more remote, and health and happiness vouchsafed a more extended reign.

It has been stated by some authority that disease is more dependent upon food and water than upon all other influences combined. This seems a very astounding assertion when we consider that life itself is also dependent upon the same. It must be understood, however, that there is a very great deal of difference between wholesome food and water and that which is capable of producing disease; and it is a fact of the utmost importance, and one which ought to be indelibly impressed upon the mind of everyone, that although both, in a pure and wholesome condition, are necessary to life and health, they also form suitable media for the cultivation and development of various pernicious agencies in the form of pathogenic bacteria and other parasitic forms which render them especially dangerous as food articles for human consumption. It is along this line of thought that I desire to ask your attention for a short time, and I would like to lay especial stress upon the desirability of securing for city populations a meat-supply and milk-supply that are as pure and wholesome as it is possible to make them.

Let us for a moment consider some of the agencies that render these articles of human food inimical to the health of the consumer. I will only mention a few, but quite sufficient to emphasize their importance as a source of danger to public health.

I will be forced to refer to some facts which may be somewhat repugnant to our finer feelings, but as they are stern realities, and there is no getting beyond them, it is only right that we should be made familiar with them. It may be all very well to be possessed with the comfortable feeling that "where ignorance is bliss 'tis folly to be wise." This may do so long as there is nothing of any particular value at stake; but when human life and health are in the balance we cannot know too much of the many untoward influences which militate against their fullest vigor; and it is only by laying bare plain facts regarding the predisposing and exciting causes of diseases that we can expect public opinion to be educated along these lines, or hope for their ultimate eradication through combined and intelligent effort.

Although the great majority of us have been in the habit, all our lives, of eating with a relish our beefsteak, our pork-chop, or perhaps some daintily spiced sausage meat, yet the minority only may have any conception that our meat animals are capable of being the bearers of a number of diseases, some of which are of a fatal character to the human family.

Let us consider one or two of the more important, and, as I have previously suggested, throw aside sentiment for the time being and view them from a matter-of-fact stand-point—the stand-point of public health.

Swine, which furnish a large proportion of our meat-supply, besides being subject to numerous other diseases, are capable of harboring, and, in fact, do harbor, several parasites which are transmitted to human beings through consumption of their flesh in an imperfectly cooked condition, which often produce serious consequences.

The muscles or flesh of this valuable food-animal furnish the habitat for an important parasite in a partially developed state, which, when partaken of with the flesh which has not undergone sufficient cooking to destroy it, comes to maturity in the human alimentary canal, and there produces what is technically known as "tæniasis," but which in plain, ordinary language means nothing more nor less than tapeworm disease.

The hog is responsible, also, for affording asylum to another parasite, which, although about the smallest of the nematode, or round worms, is mighty enough in its effects to cause embargoes to be at times placed by foreign governments upon our export hog products.

I refer here to the "trichina spiralis," which infests the muscular tissues of the hog, and when the flesh is eaten without undergoing sufficient heat the consumer becomes affected with that often fatal disease known as "trichinosis," or flesh-worm disease.

Our beef animals, too, are responsible for the transmission of tæniasis to the human consumer in a similar way to that of the hog. Such beef is popularly known as "measly beef," and, in the case of swine, "measly pork."

It may be a revelation to some to know that consumption is as prevalent relatively in cattle as it is among the human family. The germ or bacterium which produces the disease in the one case is similar to that which causes it in the other—in fact, consumption is considered to be intercommunicable.

Many of us view this subject lightly, not having devoted to it much thought; nevertheless, the question of how to prevent consumption is one which at the present time is occupying the serious attention of medical and veterinary sanitary scientists all over this country, and, in fact, throughout the entire civilized world. Only within the last month there was held in the city of London, England, a statutory general meeting of the National Association for the Prevention of Consumption and other Forms of Tuberculosis, presided over by the Earl of Derby, and at which a large number of the most eminent sanitarians of Great Britain were in attendance. The report of the Organizing Committee, which was read by the secretary, stated that they sought to enlist sympathy and coöperation by (1) the education of public opinion and the stimulation of individual initiative; (2) the influencing of Parliament, county councils, boards of guardians, chambers of agriculture, and other public authorities on matters relating to the prevention of tuberculosis; (3) the establishment throughout the kingdom of local branches of the association to be affiliated with the central office.

In the education of public opinion they suggested the circulation of pamphlets and leaflets setting forth in plain language the results of scientific investigation, public lectures by men approved by the council, addresses at congresses and other public gatherings, cooperation with other societies having for their object the promotion of public health and the establishment of open-air sanatoria for tuberculous patients, etc. I have referred to this report to show the importance that is attached in other countries to the prevention and eradication of this "great white plague." I was much struck, also, with the similarity in the objects and the method sought to enlist sympathy and coöperation in that powerful British association and in our own, as can be seen by referring to the charter of this organization.

Going back to our beef animals, we would say that in the consumption of tuberculous flesh there is danger more or less to human life, and we do not believe that anyone would relish a steak from a tuberculous animal if they were aware of the fact.

Meat although not actually diseased itself, or from an animal that may have suffered or perhaps died from some communicable disease, may yet be unfit for human consumption on account of emaciation depriving it of proper nutritive qualities, and there are certain periods in an animal's life when, although it may not be suffering from actual disease, its flesh is rendered repulsive as an article of human food.

Such, then, are a few of the conditions of our pork and beef animals through which consumers of their flesh may suffer, either from being actually diseased or from want of the proper nourishing properties of the meat.

But, besides the meat, the purity and wholesomeness of the milk-supply of our city populations is no less important a necessity.

I will not trouble you with details relative to the various causes which render milk either nocuous or innutritious, but will refer to but one condition, on account of which this "great uncooked food" becomes extremely dangerous to life, and especially that of children —I refer to tubercular milk or milk from consumptive cows.

Extensive statistics go to show that increase in the number of cases of consumption of the bowels of children (tabes mesenterica) is coincident with the time at which they are changed from their mother's milk to that of the cow. It is not the case that the bacillus of tuberculosis is found in the milk of every consumptive cow, but when the milk-vessel, the udder or bag, is affected with the disease the milk from that animal is extremely dangerous; and where a herd of dairy cattle is kept and consumption exists among them, it is possible, if not probable, that one of them may have tuberculosis of the udder, and by mixing the milk of this one cow with that of the others the whole of the supply may thereby be contaminated with tubercle bacilli.

(To be continued.)

